# Supplementary material for: Refractory Chylothorax Secondary to Sizeable Azygos Vein Hemangioma: Tailored Multimodal Treatment of a Challenging Case Report
Source: Medicina (Kaunas). 2022 Dec 31;59(1):91. doi: 10.3390/medicina59010091 (PMC9863118; doi:10.3390/medicina59010091)
Supplement: Supplementary file 1 [file medicina-59-00091-s001.zip › medicina-2112030-supplementary.pdf]

Online Supplement

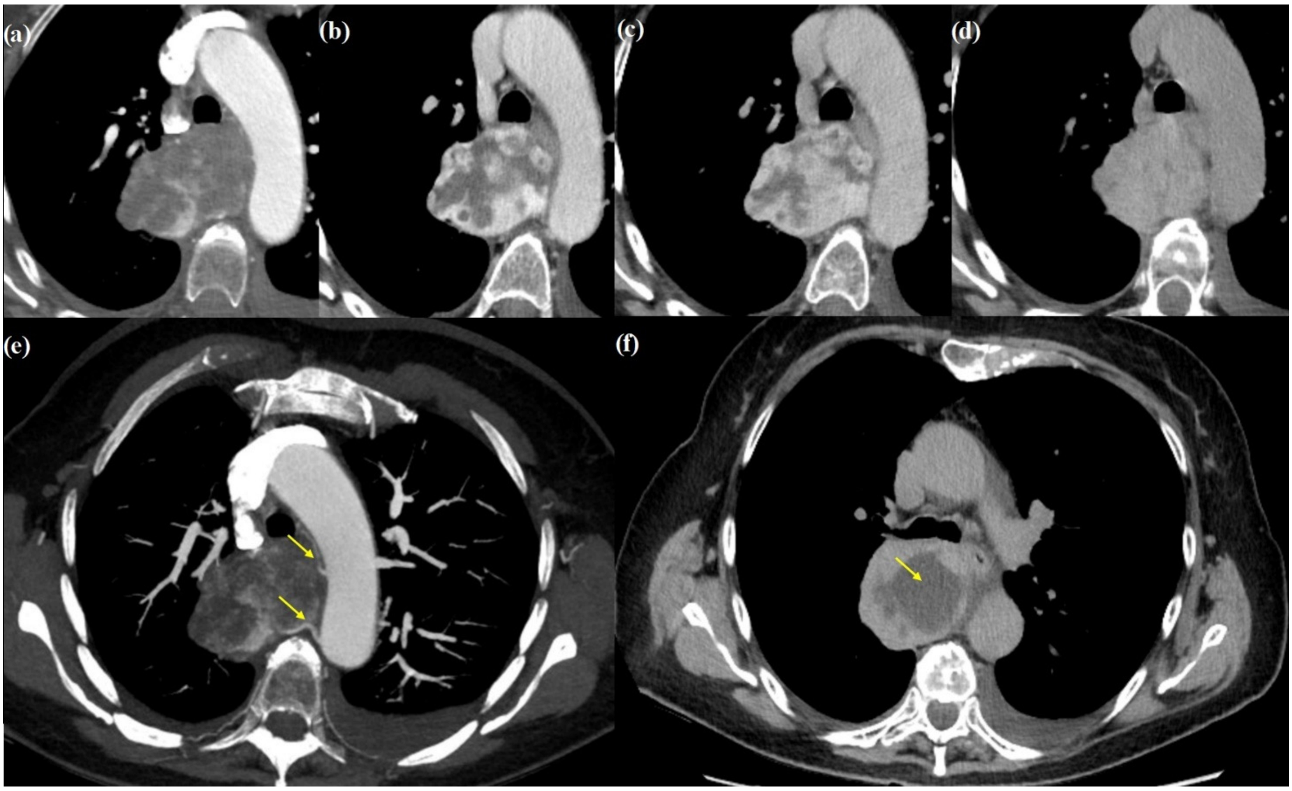

Figure S1. Enhanced CT scan study of the mediastinal mass.
(a-d) Multiphase contrast medium CT showing progressive centripetal enhancement of the mediastinal mass; (e) CT showing the two main feeding arteries (arrows) arising from the right wall of the aortic arch; (f) delayed contrast phase with the heterogeneous appearance of the mass due to a low attenuating central zone (arrow). CT: Computed Tomography.

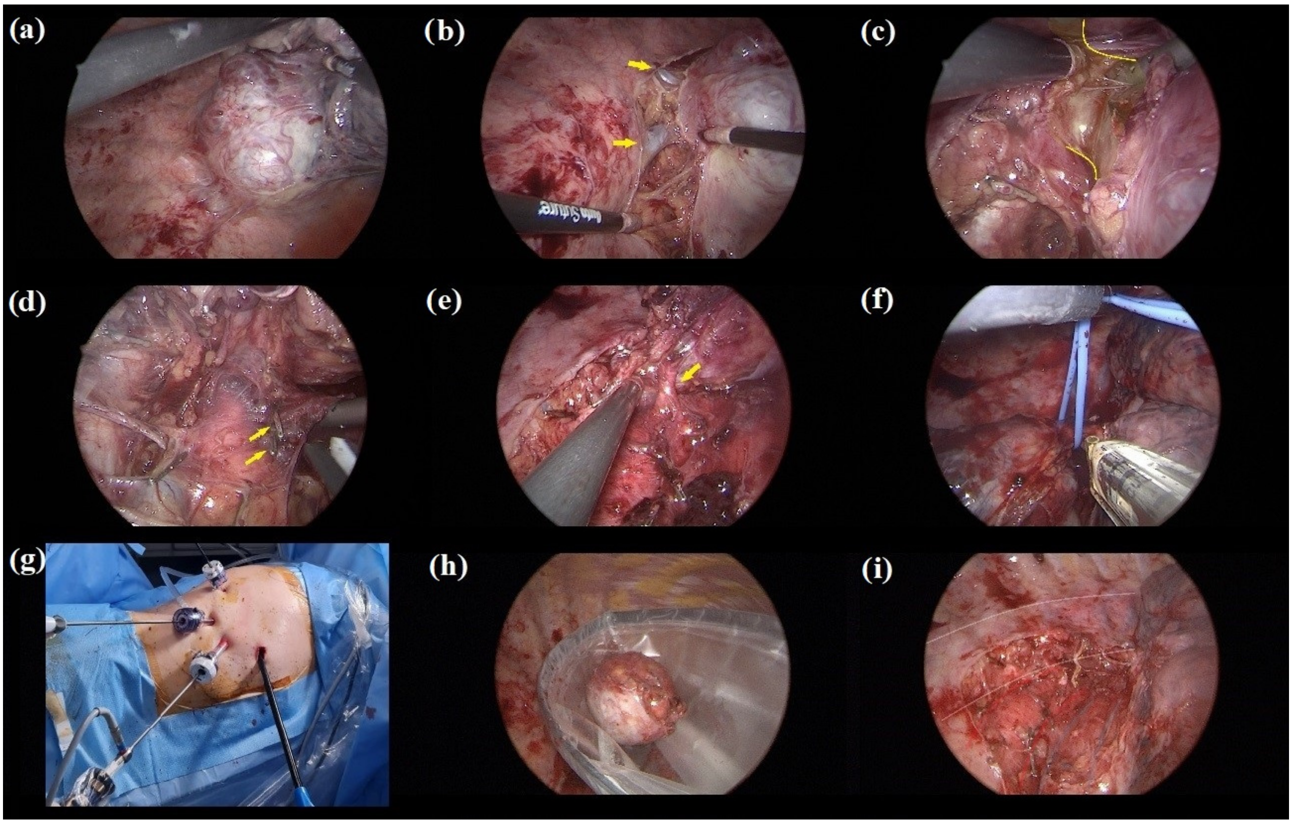

Figure S2. Video thoracoscopic surgery storyboard.

(a) Right posterior mediastinal mass with extended lung adhesions; (b) minor intercostal vein branches (arrows) are exposed; (c) identification of a sizeable intercostal vein collector of the hemangioma (yellow shape); (d-e) aortic arterial branches dissection between clip ligation (arrows); (f) leading pedicle connecting with the azygos arch being stapled; (g-h) 4-ports video-thoracoscopic positioning and hemangioma retrieval into endo-bag; (i) the resected bed of the mass exposing the vertebral bodies (white borders), the descending aorta (red borders), and the esophagus (blue borders).

Figure S3. One-year postoperative follow-up CT scan.

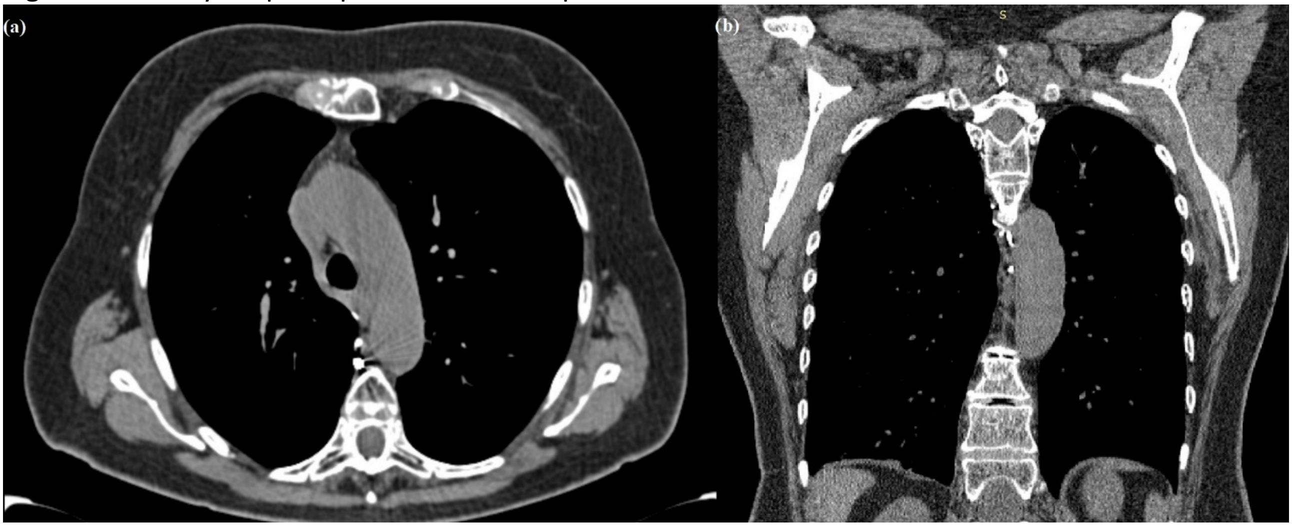

(a-b) Chest CT scan (transversal and frontal plan) shows no evidence of hemangioma recurrence or pleural effusion. CT: Chest Tomography.
